# Self-Pierce Riveting of Three Thin Sheets of Aluminum Alloy A5052 and 980 MPa Steel

**DOI:** 10.3390/ma15031010

**Published:** 2022-01-28

**Authors:** Satoshi Achira, Yohei Abe, Ken-ichiro Mori

**Affiliations:** Department of Mechanical Engineering, Toyohashi University of Technology, Toyohashi 441-8580, Japan; abe@plast.me.tut.ac.jp (Y.A.); mori@plast.me.tut.ac.jp (K.-i.M.)

**Keywords:** joining, self-pierce riveting, ultra-high strength steel sheets, aluminum alloy sheets, three sheets, plastic deformation, joint strength

## Abstract

Self-pierce riveting of three thin sheets of 980 MPa steel and 5052 aluminum alloy was performed to investigate the effect of sheet configuration on the deforming behaviors of the sheets and the rivet and joint strength. When the lower sheet was aluminum alloy, the joining range was relatively wide, i.e., the interlock hooking the rivet leg tended to be large. In the sheet configuration in which the upper and lower sheets were A5052 and the middle sheet was 980 MPa steel, the rivet leg spread out moderately and the joint without defects was obtained. In the lower 980 MPa steel sheet, fracture tended to occur due to the low ductility of the lower sheet, and the joining range was narrow with the small interlock although the three sheets were joined by an appropriate die shape. In joint strength of joined three sheets, fracture occurred in the lower-strength aluminum alloy sheet if interlocks of about 300 μm and 150 μm could be formed in the lower aluminum alloy sheet and 980 MPa steel sheet, respectively.

## 1. Introduction

The concentration of carbon dioxide, which is the cause of global warming, is increasing every year. Reduction in car body weight is one of the most important methods to reduce carbon dioxide emissions. In addition to the development of new powertrains, a significant reduction in vehicle weight is necessary to meet the stringent carbon dioxide emission regulations. Various lightweight materials such as advanced high strength steel sheets, aluminum alloy sheets, aluminum castings, and carbon fibre reinforced plastics are often introduced into car body structures. Among them, the use of aluminum alloy sheet and high strength steel sheet is increasing [1]. Reducing the weight of car body often conflicts with other missions that require increased mass, such as crashworthiness and comfort. The use of high strength steel is the most competitive way to achieve weight reduction and is often used for car body parts that require less deformation due to impact load. However, the formability and weldability of high strength steel sheet decrease as the tensile strength increases. Aluminum alloy sheet has one-third the density of steel. It also is characterized by high thermal conductivity, a natural oxide film on the surface, and a low melting point. Estimates of the amount of aluminum alloy sheet used for automobile are increasing every year, and the presence of aluminum alloy sheet in automotive materials is expected to increase in the future. The sheet configuration including these various materials are not easy to join by conventional resistance spot welding due to the difference in melting points and the problem of brittle intermetallic layer formed at the interface [2]. In addition, although two sheets are usually joined together, it is desirable to join three or more sheets to improve the design flexibility of car body panels. Lei et al. [3] conducted finite element simulations of the resistance spot welding process in joining three sheets of mild steel to investigate the transient thermal characteristics of the resistance spot welding process. Ma et al. [4] investigated the nugget formation process for three high strength steel sheets by the experiments and simulations. However, welding three sheets of dissimilar materials is even more difficult. The method of joining sheets using plastic deformation is very advantageous for dissimilar materials because it avoids difficulties in joining due to differences in melting points. Various joining methods such as friction stir welding, mechanical clinching and self-pierce riveting have been developed and used in place of the welding processes [5,6].

Fereiduni et al. [7] performed friction stir spot welding on the aluminum alloy and carbon steel to investigate the effects of tool rotation speed and dwell time on the microstructure in the joint interface and joint strength. Sato et al. [8], Feng et al. [9] and Yamamoto et al. [10] performed friction stir spot welding on aluminum alloy and galvanized steel sheets. In these studies, the two overlapping specimens, and only the upper aluminum alloy sheet was stirred, i.e., it is difficult to apply this method to multiple overlapping joints of three or more sheets.

Mechanical clinching is a method of joining sheets by locally hemming them with a punch and die without rivets. Peng et al. [11] reviewed the recent development of the clinching process. He et al. [12] investigated the clinching process using an extensible die by experiments and simulations. Chen et al. [13] joined aluminum alloy sheets by the two-steps clinching method using clinch rivets to improve the neck thickness and interlock. Réjane et al. [14] investigated the effect of the shape of the lower hole on the mechanical properties of the shear-clinched joint for aluminum alloy and steel sheets. Lee et al. [15] used a hole clinching process to joined dissimilar materials of aluminum alloy sheet and carbon fibre reinforced plastic. Abe et al. [16] prevented fracture due to concentration of deformation at the punch corner by changing the die shape in mechanical clinching of high tensile steel and aluminum alloy sheets. Not only two sheets, but also three sheets were joined by mechanical clinching, e.g., Kaðèák et al. [17] joined three steel sheets. However, the joint strength of mechanical clinched sheets obtained from these studies is not high, and it is desirable to make a joint with high joint loads.

Self-pierce riveting, is a method that allows joining without drilled holes and overlapping of sheets with gaps, is an alternative method to resistance spot welding in the joining process of car body [18]. Mori et al. [19] reviewed the mechanical joining process of dissimilar materials including self-pierce riveting. Self-pierce riveting, which mechanically joined sheets without metallic bonding, is possible to join high-tensile steel and aluminum alloy sheets at room temperature. This riveting is characterized by higher joint strength than resistance spot welding and mechanical clinching [20]. Abe et al. [21] evaluated the joining process of aluminum alloy sheet and steel sheet by self-pierce riveting using the experiments and simulations. Atzeni et al. [22] simulated the self-pierce riveting of two aluminum alloy sheets and calculated the tension-shearing strength of the joint and compared it with experimental results. Wood et al. [23] developed a finite element model of a test and measurement system at automotive crash speeds to investigate the joinability of self-pierce riveted joints of aluminum alloy sheets. Zhang [24] performed self-pierce riveting of a 1 mm thick high-strength steel and aluminum alloy sheets and investigated the effect of the sequence of riveting on the dimensional stability of the product. Jeong et al. [25] performed self-pierce riveting of high strength steel and aluminum alloy sheets and investigated the effect of sheet constraint conditions on the cross-sectional properties and joint strength.

The interlock of self-pierce riveting depends on the thickness of the sheet, the flow stress and ductility of the sheet and rivet, and the shape of the rivet and die [19]. A lot of studies were conducted to obtain the optimum joining quality for each combination of sheets. Porcaro et al. [26] conducted the tension-shearing and peel tests on the aluminum alloy sheets joined by self-pierce riveting to investigate the mechanical properties of sheets and riveted sheet shape. Ma et al. [27] investigated the effects of the rivet properties and the die shape on the deforming behaviors for the aluminum alloy and mild steel sheets. Xu [28] investigated the effect of rivet length on the cross-sectional shapes of the joint.

The studies about the optimization process of self-pierce riveting using finite element simulation were also performed. Moraes et al. [29] simulated the self-pierce riveting process of the magnesium and aluminum alloy sheets. Abe et al. [30] performed the joining of high tensile strength steel and aluminum alloy sheets and optimised die shape. Mori et al. [31] performed self-pierce riveting of ultra-high strength steel and aluminum alloy sheets and the optimum joining conditions were evaluated. However, the optimization process shown in these studies is for joining two sheets. The optimization process for severe sheet configrations such three thin sheets with large strength differences has not been clarified, i.e., it is desired to show the fundamental joinabilities.

Self-pierce riveting can be applied to joining three or more sheets [32]. Abe et al. [33] and Mori et al. [34] performed self-pierce riveting on three sheets containing high strength steel and aluminum alloy sheets. However, it was limited to conditions where the lower sheet thickness ratio was large. Mori et al. [35] conducted the joining experiments under conditions where the lower sheet thickness ratio was small, but the joining by self-pierce riveting is difficult because of the risk of cracking and the large difference in flow stress of the sheets [19,36]. In addition, self-pierce riveting has the problem that different sheet strengths affect the joinabilities, and the effect of different combinations of sheets on the deforming behaviors has not been clarified. 

In this study, self-pierce riveting of thin three sheets of 980 MPa ultra-high strength steel and aluminum alloy A5052 and finite element simulation were performed to investigate the effect of sheet configuration on the joinabilities and joint strength.

## 2. Three Sheets of Self-Pierce Riveting

### 2.1. Process and Conditions for Self-Pierce Riveting of Three Sheets

**Figure 1:**
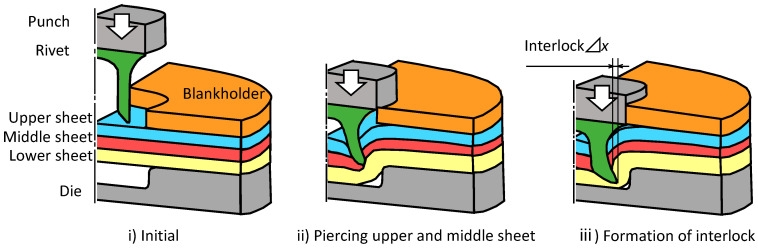
Self-pierce riveting process of three sheets.

The joining process of three sheets of self-pierce riveting is shown in Figure 1. First, the three sheets placed on top of the die are fixed with a blankholder. The rivet is then placed on top of the sheets and pushed into the sheets. After the rivet is punched through the upper and middle sheets, the rivet leg spreads out and enters the lower sheet and then three sheets are joined by an interlock.

**Figure 2:**
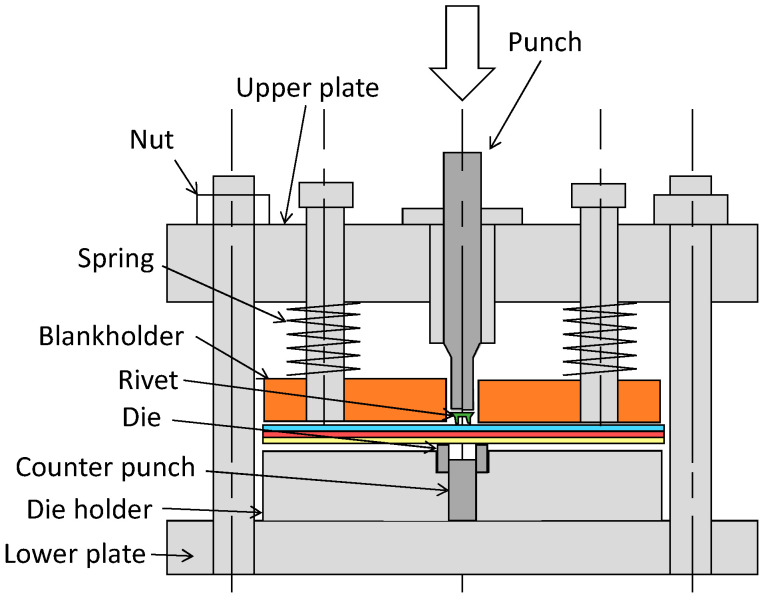
Schematic illustration of self-pierce riveting equipment.

A schematic illustration of the self-pierce riveting equipment is shown in Figure 2. Four coil springs in the upper part of the blankholder were used to apply 5 kN in blank holding force to the sheets. The die diameter *D* and the die depth *H* were changed by the die and the counter punch, respectively. The punch was pressed by a 250 kN screw driven type universal testing instrument (Autograph AGS-J, SHIMADZU Co., Kyoto, Japan). The riveting speed is 50 mm/min.

**Figure 3:**
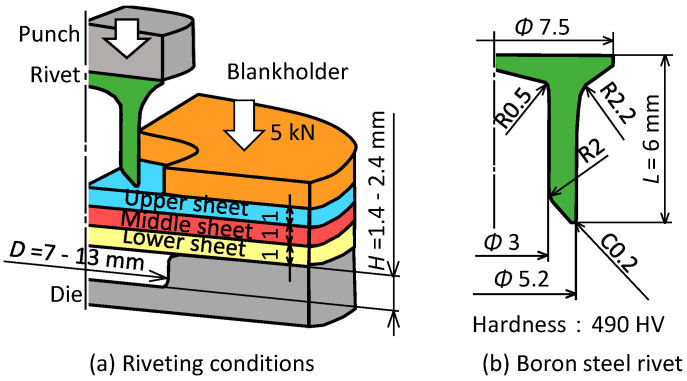
Riveting conditions of die and rivet.

The riveting conditions used in the experiments are shown in Figure 3. Aluminum alloy A5052 sheet and 980 MPa steel sheet as ultra-high strength steel sheet for car body panels were used as specimens. The material properties of the sheets obtained by the uniaxial tensile test are shown in Table 1. Three tensile test specimens were given, and the average value are shown. The sheet configuration for three-sheet joining is shown in Table 2.

**Figure 4:**
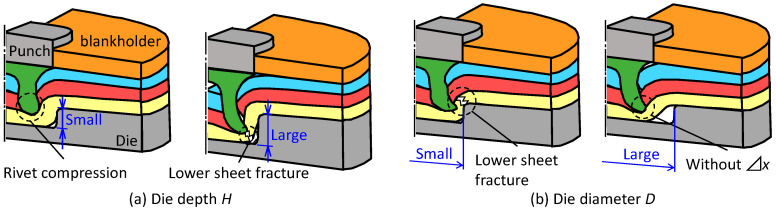
Effects of die shape on deforming behaviors of sheets and rivet.

The effect of the die shape on the deforming behaviors of the sheets and the rivet is shown in Figure 4. When the die depth is small, the rivet is compressed, and the rivet leg does not spread. When the die depth is large, fracture tends to occur because the rivet leg penetrates the lower sheet. When the die diameter is small, the deformation concentrates at the die corner, causing the lower sheet to fracture. When the die diameter is large, the interlock cannot be formed because the reaction force from the die wall cannot be obtained.

### 2.2. Simulation Conditions and Results

The commercial finite element code LS-DYNA was used for the simulation to predict the deforming behaviors of the sheets and rivet. The conditions of simulation are shown in Table 3. A dynamic explicit method was used. The symmetric conditions were applied. The punch, blankholder and die were rigid bodies, and the sheets were isotropic elasto-plastic materials, taking into account the strain effect using the power hardening law. The rivet and the sheet were divided by a quadrilateral solid element, and the element size was 0.1 mm × 0.1 mm. In the simulation, remeshing of the elements was automatically applied to increase the calculating accuracy. When the size of element in the sheet fell below a certain value, the element was removed. The certain value of the element size was set to the minimum thickness before fracture in the experiment, which was 0.1 mm for aluminum alloy sheet and 0.35 mm for 980 MPa steel sheet, respectively. The coulomb friction was assumed for all interfaces among sheets, tools and rivets, and the coefficient of friction was 0.20. The material properties of the sheets in the simulation are shown in Table 4.

**Figure 5:**
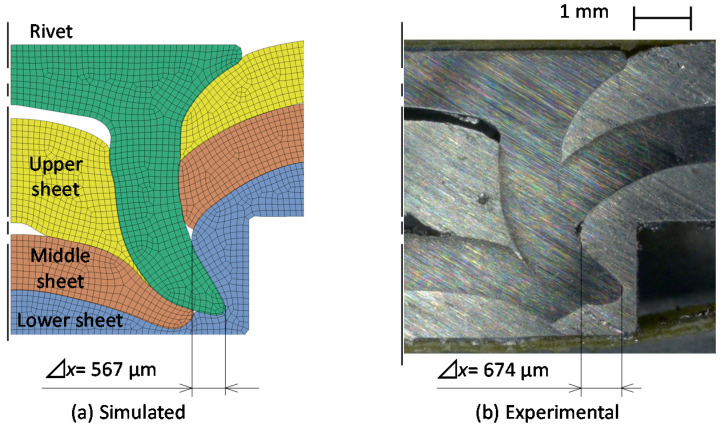
Comparison of simulated cross-sectional shapes with experimental one.

The simulated and experimental cross-sectional shapes are shown in Figure 5. The simulated cross-sectional shape is similar to the experimental one, although the experimental interlock is larger than the simulated one.

### 2.3. Joining Requirements

**Figure 6:**
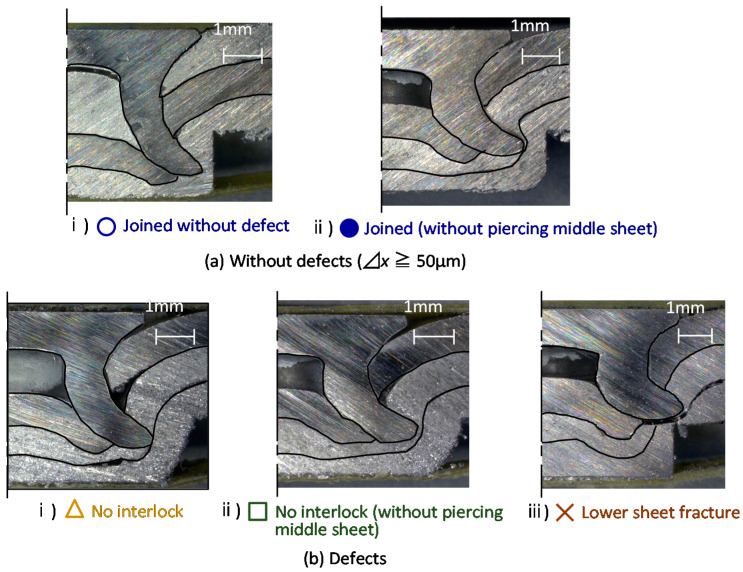
Cross-sectional shapes in experiment.

The cross-sectional shapes of sheets in the experiment are shown in Figure 6. In this study, a joint without defects was defined as the formation of 50 μm or more in length of the interlock without fracture in the lower sheet as shown in Figure 6a. The conditions that did not satisfy these requirements were defined as defects: for example, no interlock and the lower sheet fracture as shown in Figure 6b.

## 3. Three Sheets Joining of 980 MPa Steel and Aluminum Alloy

### 3.1. Three Sheets Joining in Lower Aluminum Alloy

**Figure 7:**
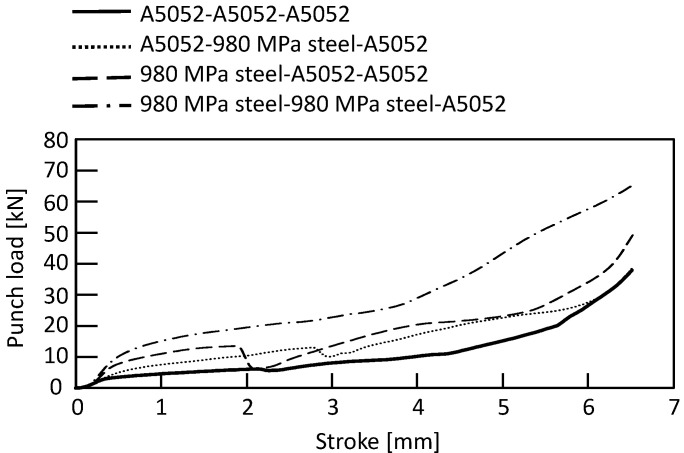
Punch-load-stroke curves in lower aluminum alloy sheet for *D* = 9 mm and *H* = 1.8 mm obtained from experiment.

The punch-load-stroke curves for *D* = 9 mm and *H* = 1.8 mm obtained from the experiment are shown in Figure 7. The punch load increased as the stroke increased. The punch load was higher as the number of steel sheets increased. The punch load decreased when the rivet punched through the sheet.

**Figure 8:**
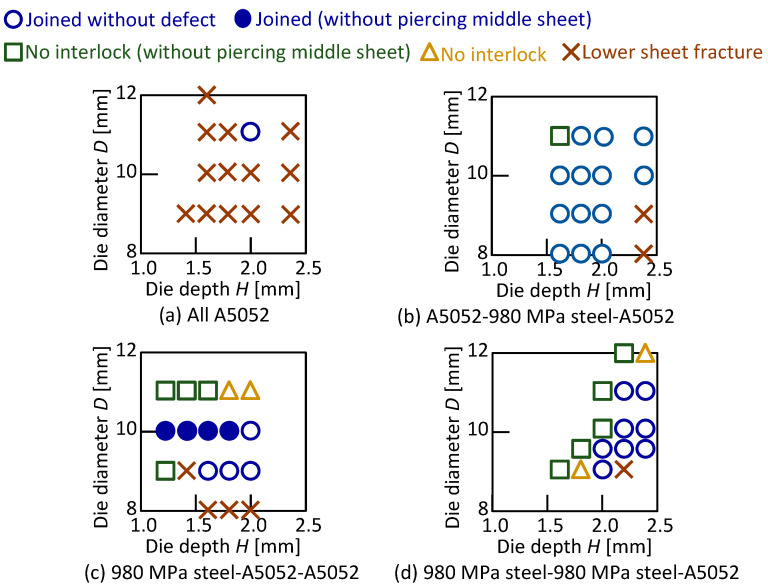
Joining range in lower aluminum alloy sheet.

The joining results in the lower aluminum alloy sheet are shown in Table 5, and the joining range is shown in Figure 8. A relatively wide joining range was obtained for the lower aluminum alloy sheet, and the widest range was obtained for A5052-980 MPa steel-A5052. The joining range in the configuration of the upper 980 MPa steel sheet tended to be narrow. The joining range of the three aluminum alloy sheets was limited.

**Figure 9:**
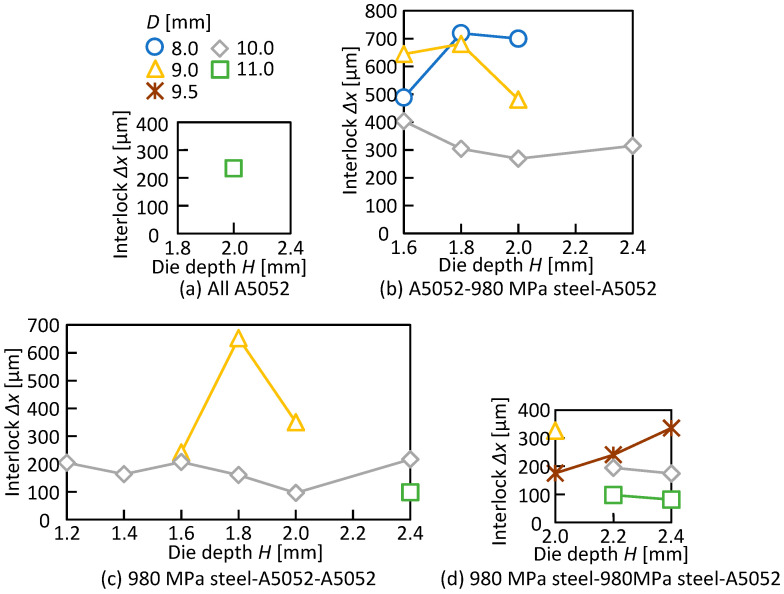
Relationship between die shape and interlock.

The relationship between the die shape and the interlock in the joined three sheets is shown in Figure 9. In A5052-980 MPa steel-A5052, which showed the widest joining range, the interlock of 250 μm or more was obtained in all conditions. The interlock decreased in both the 980 MPa steel-A5052-A5052 and 980 MPa steel-980 MPa steel-A5052 than that of A5052-980 MPa steel-A5052 under the same die shape.

**Figure 10:**
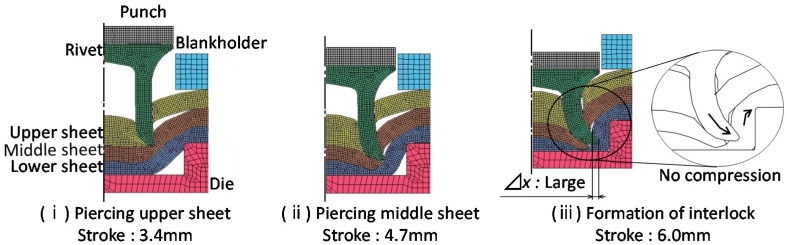
Cross-sectional shapes in simulation for *D* = 9 mm and *H* = 1.8 mm and A5052-980 MPa steel-A5052.

The simulated cross-sectional shapes in the lower aluminum alloy sheet for A5052-980 MPa steel-A5052 are shown in Figure 10. The amount of rivet compression was small even when the rivet entered the lower sheet because of the low strength of the lower sheet, and the rivet tended to easily form the interlock. Thus, it is considered that a relatively wide joining range was obtained in the lower aluminum alloy sheet.

**Figure 11:**
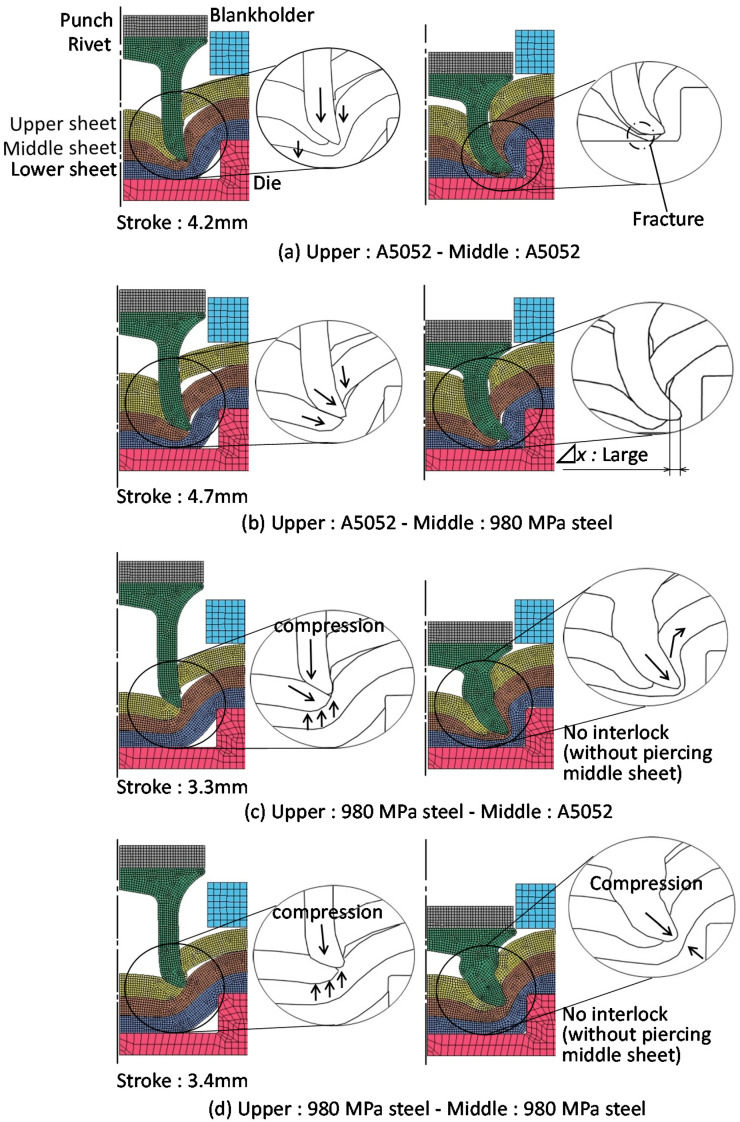
Effect of strength of upper and middle sheets on deforming behaviors of sheets and rivet for *D* = 9 mm, *H* = 1.8 mm and lower A5052 sheet.

The effects of the upper and middle sheets on the deforming behaviors are shown in Figure 11. In Figure 11a, the rivet leg did not spread in the soft three A5052 sheets, and the rivet tip pierced the lower sheet as shown in Figure 8a. The rivet leg tended to spread moderately upon the penetration of the middle sheet due to the high strength of the middle sheet in Figure 11b. In the experiments as shown in Figure 8b and Figure 9b, the wide joint range and the large interlock were obtained. In Figure 11c,d, since the upper sheet was of high strength, the rivet leg tended to be highly compressed, resulting in unpenetrated middle sheets. The result in the experiment as shown in Figure 8 was similar. For the sheet configurations in Figure 11c,d, the joining range can be obtained by setting the die shape parameters *D* and *H* appropriately.

### 3.2. The Tension-Shearing Test in Lower Aluminum Alloy

**Figure 12:**
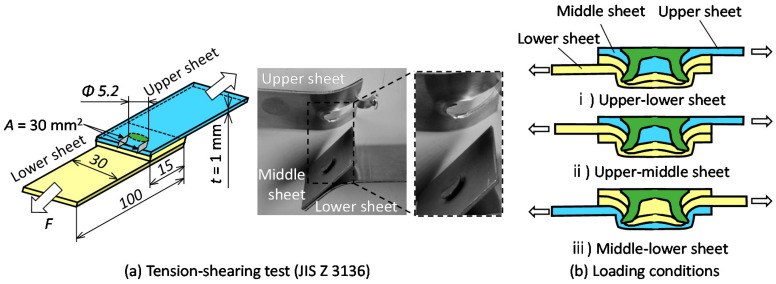
Measuring conditions of joint strength.

The joining strength was measured by a tension-shearing test. The measuring conditions of the joint strength are shown in Figure 12. The tension-shearing test was performed based on the test for the resistance spot welded joints [37]. The specimens were selected according to the JIS Z3136. A single joint was made at the center of the overlapped sheets as shown in Figure 12a. A 50 kN screw driven type universal testing instrument (Autograph AGS-J, SHIMADZU Co., Kyoto, Japan) was used for this test, and the tensile speed was 10 mm/min. Since the specimens were in triplicate, there were three different loading combinations: the upper and lower, the upper and middle, and the middle and lower sheets as shown in Figure 12b. The tension-shearing load *F*_s_ in fracture by shearing in the area of the rivet outer diameter to the sheet edge is expressed by the following Equation (1).

(1) $$F_{s}=Ak\sigma_{a}$$

where *A* is the cross-sectional area from the rivet outer diameter to the sheet edge, *σ*_a_ is the tensile strength of the aluminum alloy sheet, and *k* is the factor of shear strength. The values in Table 1, *k* = $\frac{1}{\sqrt{3}}$ and *A* = 30 mm^2^ were substituted in the Equation (1);

$$F_{s}=30\;\times \;\frac{1}{\sqrt{3}}\;\times \;275=4.76\;[\mathrm{kN}]$$

The tension-shearing load *F*_r_ at which the rivet leg is fractured by shearing is expressed by the following equation.

(2) $$F_{r}=\frac{\pi }{4}(d_{o}{}^{2}-d_{i}{}^{2})k\sigma_{r}$$

where, the tensile strength of a rivet is *σ*_r_ and the external and internal diameters of the rivet leg are *d_o_* and *d_i_*, respectively. The values in Figure 3b and *σ*_r_ = 1600 MPa were substituted in the Equation (2).

$$F_{r}=\frac{\pi }{4}(5.2^{2}-3^{2})\;\times \;\frac{1}{\sqrt{3}}\;\times \;1600=13.1\;[\mathrm{kN}]$$

thus, the tension-shearing load *F*_s_, assuming fracture of the aluminum alloy sheet, is smaller than the load *F*_r_.

**Figure 13:**
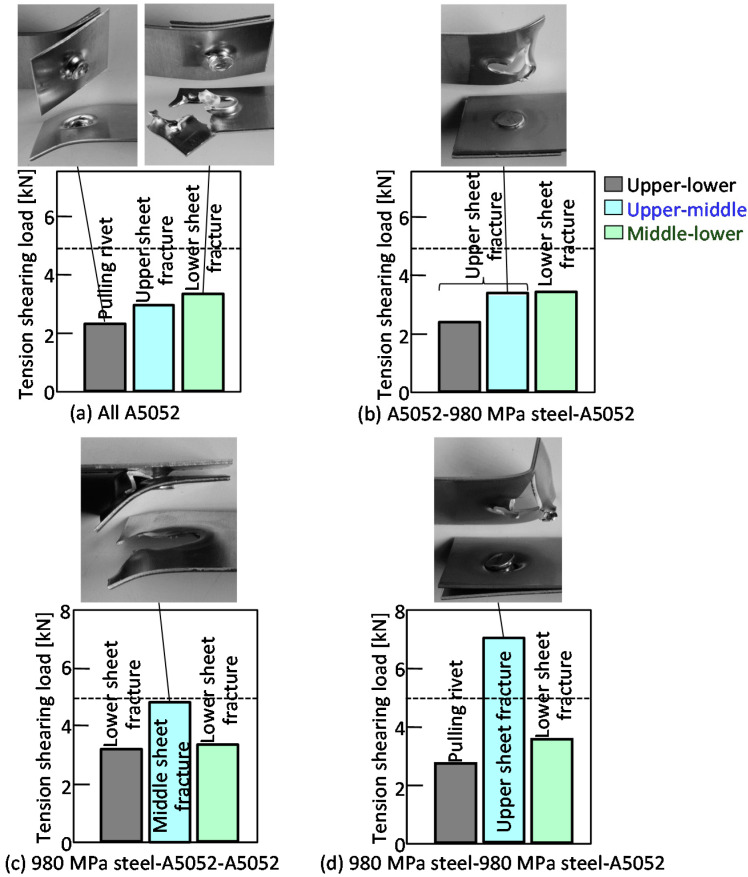
Tension-shearing load in lower aluminum alloy sheet.

The tension-shearing load in the lower aluminum alloy sheet is shown in Figure 13. In the case of configurations containing low strength aluminum alloy sheets, fracture occurred mostly in the aluminum alloy sheet. In the upper-lower sheet tensile test shown in Figure 13a,b, pulling of the rivet occurred before fracture in the aluminum alloy sheet. The maximum tension-shearing load under the conditions where fracture occurred in the aluminum alloy sheet was about 3.0 kN, which was about 63% of the Equation (1). However, this was the load at which the rivet was pulled out before the aluminum alloy sheet was completely fractured, as shown in the deformed joint. It was considered that the measured load was lower than the Equation (1). In the upper-middle sheet in Figure 13c, the aluminum alloy sheet completely fractured, and the load was 4.7 kN, which was equivalent to the Equation (1). In the upper-middle sheet in Figure 13d, the load was as high as 7.0 kN even when the pulling rivet occurred, because the tension-shearing load was applied between the steel sheets.

**Figure 14:**
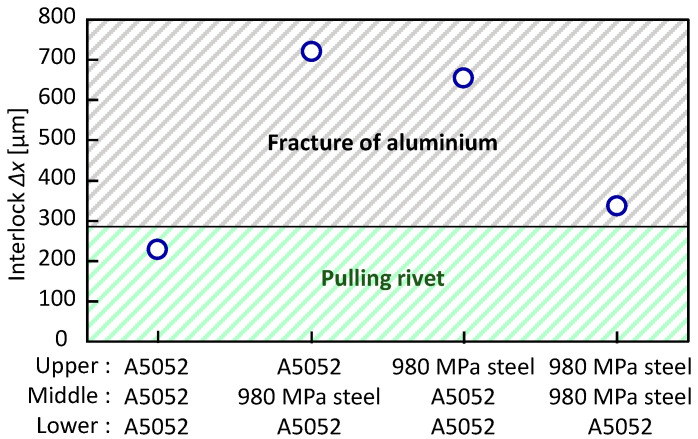
Relationship between configuration and interlock in lower aluminum alloy sheet.

The relationship between the configuration and the interlock in the lower aluminum alloy sheet is shown in the Figure 14. In the three aluminum alloy sheets, the rivet was pulled out from the lower sheet. Of the three sheet configurations in which the lower aluminum alloy sheet was fractured, 980 MPa steel-980 MPa steel-A5052 had the lowest interlock, i.e., about 300 μm in interlock was sufficient in the lower aluminum alloy sheet.

### 3.3. Three Sheets Joining in Lower 980 MPa Steel

**Figure 15:**
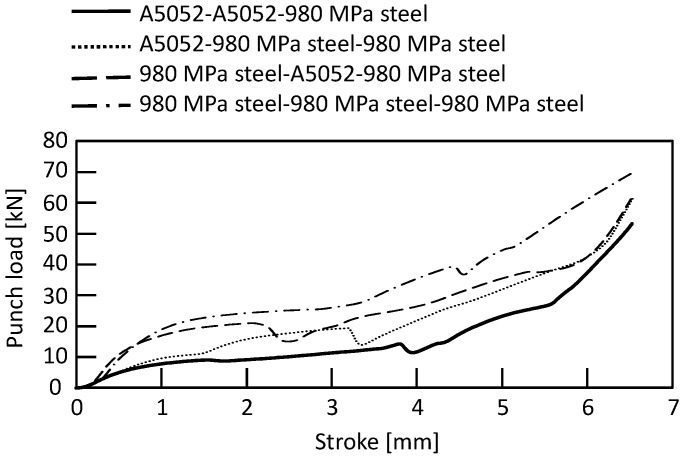
Punch-load-stroke curves in lower 980 MPa steel sheet for *D* = 9 mm and *H* = 1.8 mm obtained from experiment.

The punch-load-stroke curves in the lower 980 MPa steel sheet for *D* = 9 mm and *H* = 1.8 mm obtained from the experiment are shown in Figure 15. Compared with the lower aluminum alloy sheet, the maximum punch load was larger for all sheet configurations.

**Figure 16:**
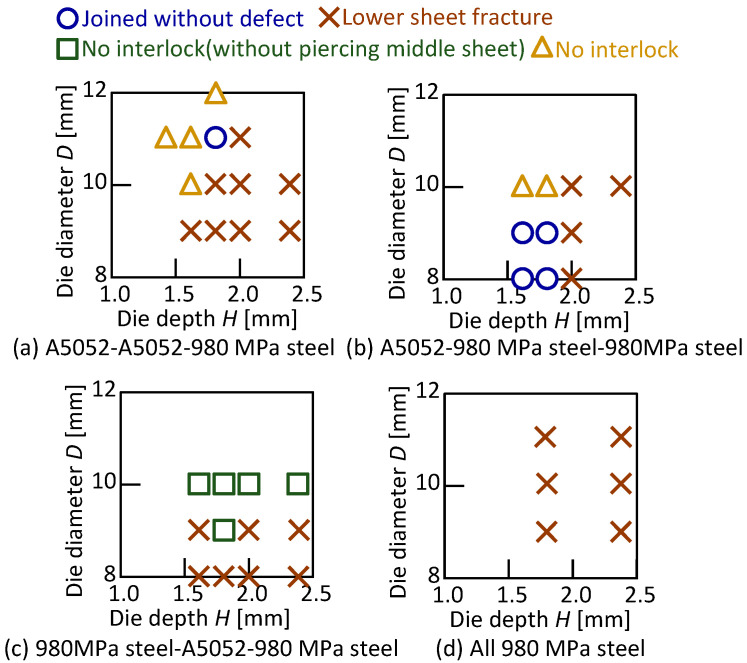
Joining range in lower 980 MPa steel sheet.

The joining results in the lower 980 MPa steel sheet are shown in Table 6, and then the joining range is shown in Figure 16. In Figure 16a,b, the joining conditions without defect were obtained, but the joining range was narrower than for the lower aluminum alloy sheet with the same configuration of upper and middle. The sheet configuration in Figure 16c was not joined because the rivet leg tended to spread to the die corner, causing the lower sheet to fracture. In Figure 16d, the lower sheet fractured due to low ductility of the sheets in all conditions. Among the eight types of sheet configurations including Figure 14, A5052-980 MPa steel-A5052 was the best configuration to join because it provided the widest joining range by moderately spreading the rivet leg so that the large interlock was obtained.

**Figure 17:**
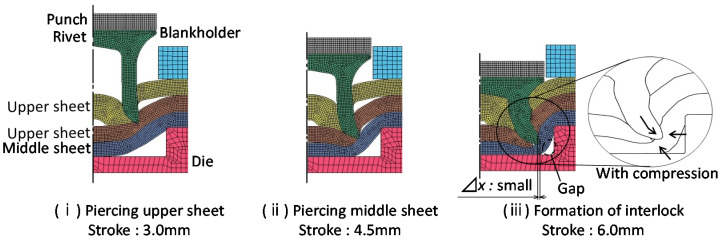
Cross-sectional shapes of sheets and rivet in simulation for *D* = 9 mm, *H* = 1.8 mm and A5052-980 MPa steel-980 MPa steel.

The cross-sectional shapes of the sheets and rivet in the simulation for A5052-980 MPa steel-980 MPa steel are shown in Figure 17. The rivet leg tended to be compressed because the middle and lower sheets were high strength whereas the rivet penetrated the upper sheet easily. The deformations of the middle and lower sheets became small, and the interlock tended to be small.

### 3.4. The Tension-Shearing Test in Lower 980 MPa Steel

**Figure 18:**
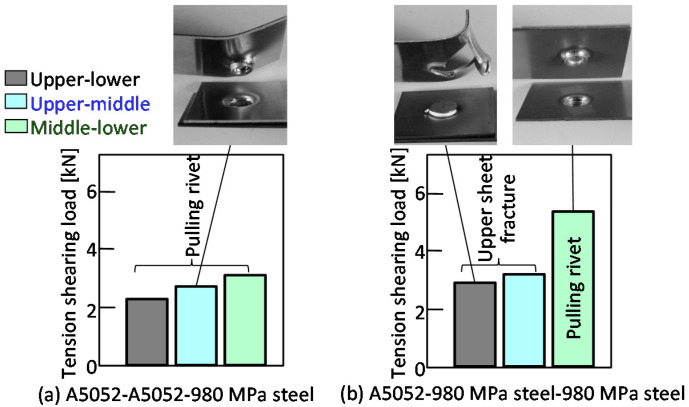
Tension-shearing load in lower 980 MPa sheet.

The tension-shearing load in the lower 980 MPa steel sheet is shown in Figure 18. In Figure 18a, the pulling rivet occurred before the fracture aluminum alloy sheet in all conditions. The maximum tension-shearing load was about 2 to 3 kN, which was about 42 to 63% of the Equation (1). In Figure 18b, fracture occurred in the low strength aluminum alloy sheet. The maximum tension-shearing load was about 3.0 kN, it was similar to the load in the lower aluminum alloy sheet. In the middle-lower sheet, the tension-shearing load was as high as 5.3 kN, even when the pulling rivet occurred, because the tension-shearing load was applied between the steel sheets.

**Figure 19:**
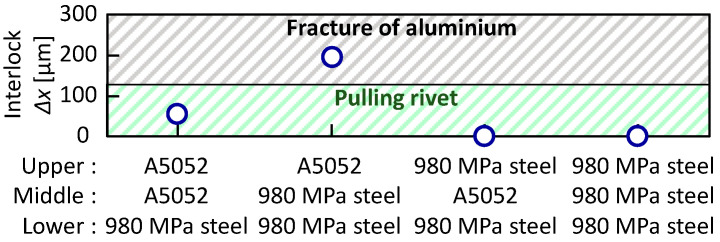
Relationship between configuration and interlock in lower 980 MPa steel sheet.

The relationship between the sheet configuration and the interlock in the lower 980 MPa steel sheet is shown in Figure 19. The interlock tended to be smaller in all configurations than that in the lower aluminum alloy sheet in Figure 14. The interlock in A5052-980 MPa steel-980 MPa steel, for which without defect conditions were obtained, was 198 μm. Fracture occurred in the aluminum alloy sheet, except for the condition where the steel sheets were given a tension. The formation of about 150 μm in interlock is sufficient because fracture occurs in the low strength aluminum alloy sheet in the lower 980 MPa steel sheet.

## 4. Conclusions

In this study, the deforming behaviors of three thin sheets and a rivet—including the ultra-high strength steel and aluminum alloy sheets—were investigated by self-pierce riveting and finite element simulation. The results obtained are shown below;

(1) When the lower sheet was the aluminum alloy, the joint range was relatively wide, and the interlock tended to be large;
(2) In the lower 980 MPa steel sheet, A5052-A5052-980 MPa steel and A5052-980 MPa steel-980 MPa steel were joined by selecting an appropriate die shape. Due to the low ductility and high flow stress of the lower sheet, fracture tended to occur, resulting in a narrower joining range where the interlock was not large;
(3) Among the eight types of sheet configurations, A5052-980 MPa steel-A5052 was the best configuration to join because it provided the widest joining range by moderately spreading the rivet leg so that a large interlock was obtained;
(4) In the tension-shearing test, fracture occurred in the lower-strength aluminum alloy sheet if interlocks of about 300 μm and 150 μm could be formed in the lower aluminum alloy sheet and 980 MPa steel sheet, respectively.

## Figures and Tables

**Table 1:** Material properties of sheets.

Sheet	Sheet Thickness [mm]	Tensile Strength [MPa]	Elongation [%]	Reduction in Area [%]
A5052	1.05	275	25	65
980 MPa steel	1.05	1002	14	35

**Table 2:** Configuration of sheets used in joining experiment.

Sheet	1	2	3	4	5	6	7	8
Upper	A5052	A5052	980 MPa steel	980 MPa steel	A5052	A5052	980 MPa steel	980 MPa steel
Middle	A5052	980 MPa steel	A5052	980 MPa steel	A5052	980 MPa steel	A5052	980 MPa steel
Lower	A5052	A5052	A5052	A5052	980 MPa steel	980 MPa steel	980 MPa steel	980 MPa steel

**Table 3:** Conditions of simulation.

Solver	LS-DYNA
Simulation method	Dynamic explicit
Model	Axial symmetry
Plastic deformation	Isotropy
Yield criterion	Von Mises
Hardening equation	$\bar{\sigma }=K{\bar{\varepsilon }}^{n}$
Coefficient of friction	0.2

**Table 4:** Material properties of sheets in simulation.

Sheet	Young’s Modulus [GPa]	*K*-Value ^※^ [MPa]	*n*-Value ^※^ [-]
A5052	70.3	550	0.32
980 MPa steel	210	1406	0.13

**^※^** $\bar{\sigma }=K{\bar{\varepsilon }}^{n}$.

**Table 5:** Joining results in lower aluminum alloy sheets.

Upper Sheet	Middle Sheet	Die Depth *H* [mm]	Die Diameter *D* [mm]	Joining Results
A5052	A5052	1.4	9.0	×
A5052	A5052	1.6	9.0	×
A5052	A5052	1.6	10.0	×
A5052	A5052	1.6	11.0	×
A5052	A5052	1.6	12.0	×
A5052	A5052	1.8	9.0	×
A5052	A5052	1.8	10.0	×
A5052	A5052	1.8	11.0	×
A5052	A5052	2.0	9.0	×
A5052	A5052	2.0	10.0	×
A5052	A5052	2.0	11.0	○
A5052	A5052	2.4	9.0	×
A5052	A5052	2.4	10.0	×
A5052	A5052	2.4	11.0	×
A5052	980 MPa Steel	1.6	8.0	○
A5052	980 MPa Steel	1.6	9.0	○
A5052	980 MPa Steel	1.6	10.0	○
A5052	980 MPa Steel	1.6	11.0	□
A5052	980 MPa Steel	1.8	8.0	○
A5052	980 MPa Steel	1.8	9.0	○
A5052	980 MPa Steel	1.8	10.0	○
A5052	980 MPa Steel	1.8	11.0	○
A5052	980 MPa Steel	2.0	8.0	○
A5052	980 MPa Steel	2.0	9.0	○
A5052	980 MPa Steel	2.0	10.0	○
A5052	980 MPa Steel	2.0	11.0	○
A5052	980 MPa Steel	2.4	8.0	×
A5052	980 MPa Steel	2.4	9.0	×
A5052	980 MPa Steel	2.4	10.0	○
A5052	980 MPa Steel	2.4	11.0	○
980 MPa Steel	A5052	1.2	9.0	□
980 MPa Steel	A5052	1.2	10.0	●
980 MPa Steel	A5052	1.2	11.0	□
980 MPa Steel	A5052	1.4	9.0	×
980 MPa Steel	A5052	1.4	10.0	●
980 MPa Steel	A5052	1.4	11.0	□
980 MPa Steel	A5052	1.6	8.0	×
980 MPa Steel	A5052	1.6	9.0	○
980 MPa Steel	A5052	1.6	10.0	●
980 MPa Steel	A5052	1.6	11.0	□
980 MPa Steel	A5052	1.8	8.0	×
980 MPa Steel	A5052	1.8	9.0	○
980 MPa Steel	A5052	1.8	10.0	●
980 MPa Steel	A5052	1.8	11.0	△
980 MPa Steel	A5052	2.0	8.0	×
980 MPa Steel	A5052	2.0	9.0	○
980 MPa Steel	A5052	2.0	10.0	○
980 MPa Steel	A5052	2.0	11.0	△
980 MPa Steel	980 MPa Steel	1.6	9.0	□
980 MPa Steel	980 MPa Steel	1.8	9.0	△
980 MPa Steel	980 MPa Steel	1.8	9.5	□
980 MPa Steel	980 MPa Steel	2.0	9.0	○
980 MPa Steel	980 MPa Steel	2.0	9.5	○
980 MPa Steel	980 MPa Steel	2.0	10.0	□
980 MPa Steel	980 MPa Steel	2.0	11.0	□
980 MPa Steel	980 MPa Steel	2.2	9.0	×
980 MPa Steel	980 MPa Steel	2.2	9.5	○
980 MPa Steel	980 MPa Steel	2.2	10.0	○
980 MPa Steel	980 MPa Steel	2.2	11.0	○
980 MPa Steel	980 MPa Steel	2.2	12.0	□
980 MPa Steel	980 MPa Steel	2.4	9.5	○
980 MPa Steel	980 MPa Steel	2.4	10.0	○
980 MPa Steel	980 MPa Steel	2.4	11.0	○
980 MPa Steel	980 MPa Steel	2.4	12.0	△

**Table 6:** Joining results in lower 980 MPa steel sheet.

Upper Sheet	Middle Sheet	Die Depth *H* [mm]	Die Diameter *D* [mm]	Joining Results
A5052	A5052	1.4	11.0	△
A5052	A5052	1.6	9.0	×
A5052	A5052	1.6	10.0	△
A5052	A5052	1.6	11.0	△
A5052	A5052	1.8	9.0	×
A5052	A5052	1.8	10.0	×
A5052	A5052	1.8	11.0	○
A5052	A5052	1.8	12.0	△
A5052	A5052	2.0	9.0	×
A5052	A5052	2.0	10.0	×
A5052	A5052	2.0	11.0	×
A5052	A5052	2.4	9.0	×
A5052	A5052	2.4	10.0	×
A5052	980 MPa Steel	1.6	8.0	○
A5052	980 MPa Steel	1.6	9.0	○
A5052	980 MPa Steel	1.6	10.0	△
A5052	980 MPa Steel	1.8	8.0	○
A5052	980 MPa Steel	1.8	9.0	○
A5052	980 MPa Steel	1.8	10.0	△
A5052	980 MPa Steel	2.0	8.0	×
A5052	980 MPa Steel	2.0	9.0	×
A5052	980 MPa Steel	2.0	10.0	×
A5052	980 MPa Steel	2.4	10.0	×
980 MPa Steel	A5052	1.6	8.0	×
980 MPa Steel	A5052	1.6	9.0	×
980 MPa Steel	A5052	1.6	10.0	□
980 MPa Steel	A5052	1.8	8.0	×
980 MPa Steel	A5052	1.8	9.0	□
980 MPa Steel	A5052	1.8	10.0	□
980 MPa Steel	A5052	2.0	8.0	×
980 MPa Steel	A5052	2.0	9.0	×
980 MPa Steel	A5052	2.0	10.0	□
980 MPa Steel	A5052	2.4	8.0	×
980 MPa Steel	A5052	2.4	9.0	×
980 MPa Steel	A5052	2.4	10.0	□
980 MPa Steel	980 MPa Steel	1.6	9.0	×
980 MPa Steel	980 MPa Steel	1.6	10.0	×
980 MPa Steel	980 MPa Steel	1.6	11.0	×
980 MPa Steel	980 MPa Steel	2.4	9.0	×
980 MPa Steel	980 MPa Steel	2.4	10.0	×
980 MPa Steel	980 MPa Steel	2.4	11.0	×

## Data Availability

All data are available from the corresponding author upon reasonable request.
